# Explicit Mental Health Messaging Promotes Serious Video Game Selection in Youth With Elevated Mental Health Symptoms

**DOI:** 10.3389/fpsyg.2018.01837

**Published:** 2018-10-02

**Authors:** Marlou Poppelaars, Aniek Wols, Anna Lichtwarck-Aschoff, Isabela Granic

**Affiliations:** Radboud University, Behavioural Science Institute, Nijmegen, Netherlands

**Keywords:** serious video games, mental health, messaging, affect, intrinsic motivation, depressive symptoms, anxiety symptoms, stress symptoms

## Abstract

Serious games aimed at promoting well-being in youth have promising effects and potential for far-reaching impact. Considering that most mental health disorders remain untreated in youth, therapeutic games may be most valuable when they are aimed at untreated youth with internalizing symptoms. However, when targeting youth outside of a clinical setting, the first impression of therapeutic video games may determine whether and how a game is played. Thus, understanding the influence of messaging used in the promotion of therapeutic games on game choice and experience is critical. The current study examined two alternatives in promoting mental health games: one included explicit mental health messaging (e.g., learn to manage stress) and the other was a stealth promotion that did not mention mental health but highlighted the entertainment value. Young adults with mild to severe internalizing mental health symptoms (i.e., depressive, anxiety, and stress symptoms) were shown two distinct trailer designs, with random assignment determining which design held which message. Participants (*n* = 129, *M_age_* = 21.33, *SD_age_* = 3.20), unaware that both trailers promoted the same commercial video game, were 3.71 times more likely to choose what they believed was the mental health game. Additionally, an unforeseen difference in the attractiveness of the two trailer designs resulted in participants being 5.65 times more likely to select the mental health game promoted in one trailer design over the other. Messaging did not influence game experience (i.e., gameplay duration, autonomy, competence, intrinsic motivation and affect). Exploratory analyses indicated that game experience, but not game choice, was influenced by symptom severity, symptom type and the interaction between symptom severity and messaging. The present study suggests that explicit mental health messages attract youth with mental health symptoms. Ultimately, youth may be empowered to seek out mental health games if they are promoted properly, allowing for far-reaching positive influences on well-being. Toward this aim, future research is needed on the game selection process, addressing underlying motivations, the balance between explicit health and entertainment messaging, and multiple interacting influences on game selection (e.g., promotion and peers).

## Introduction

Video games are immensely popular among youth (Lenhart et al., 2008; Entertainment Software Association, 2017). Consequently, the idea to capitalize on this popularity and to use games to teach youth skills is thriving (Wilkinson et al., 2008; Sardi et al., 2017; Dias et al., 2018). Serious games are designed to teach knowledge, skill or behavior change and may be used to promote mental well-being in youth (Lau et al., 2017). Mental health games can be offered as tools to supplement standard therapy or as a replacement of school-based prevention programs (e.g., Fernández-Aranda et al., 2012; Schoneveld et al., 2018). However, the potential for impact may be far greater. Mental health games may be used to promote overall well-being in the general population, to offer light interventions to people with mild mental health symptoms or to reach individuals with clinical disorders who are not seeking professional help. Whether or not serious games can reach such diverse populations may in part be determined by how games are presented. Therefore, the aim of the current study was to test how messaging used to promote therapeutic games affects the game choice of youth and their experience of gameplay outside of a therapeutic context. Our focus in the current study was on youth as they are both avid consumers of video games (Entertainment Software Association, 2017) and particularly vulnerable to the development of psychological disorders (Merikangas et al., 2010).

The idea to capitalize on the popularity of video games stems from the attraction and engagement of video games. The potential that using games for therapeutic purposes holds has researchers and therapists excited for several reasons. Specifically, games may: (1) get youth motivated to learn skills; (2) attract and retain youth in therapy programs; (3) help youth persevere throughout therapy’s strenuous process, similar to persisting in a difficult game level; (4) help youth realistically practice and (5) facilitate internalization and generalization of new skills (see e.g., Granic et al., 2014; Buday, 2015; Fleming et al., 2017). Although immensely challenging, creating games in which the therapeutic aim and engagement of game design enhance each other has some precedence. One example is *MindLight*, an effective anxiety reduction game that successfully evokes anxiety during gameplay, trains regulation of these anxious feelings, and engages children to the level that they would recommend the game to others as much as they would recommend a commercial game (Schoneveld et al., 2016, 2018). With this engagement potential of therapeutic games, it would be a shame to have mental health games only be played as part of a therapy protocol rather than reaching billions of people who enjoy commercial video games (Meeker, 2017).

While the first target population for serious games may be youth already in therapy, for whom serious games could supplement a therapist-led intervention, serious games can also be aimed at youth not (currently) in therapy. Specifically, a particularly interesting target population for serious games are youth with mild mental health issues, because: (1) their symptoms may be mild enough to be alleviated through an intervention outside of a clinical setting and (2) prevention research has repeatedly shown larger effect sizes for prevention targeted at risk groups compared to universal prevention (Horowitz and Garber, 2006; Stockings et al., 2016).

Moreover, people with acute mental health problems who nevertheless do not seek help is a third target population for serious games. A wide range of studies demonstrates that professional help-seeking is low in youth, with only about a quarter to a third of youth seeking help for diagnosable mood and anxiety disorders (Alonso et al., 2004; Merikangas et al., 2011). Perceived stigma is one important barrier that prevents youth from seeking help (Clement et al., 2015). Youth tend to feel self-conscious and embarrassed about seeking help from professionals and are concerned about confidentiality (Gulliver et al., 2010). Serious games may relieve the most pressing distress of these youth and/or aim to encourage youth to seek professional help. Thus, although serious games may target a range of populations, they may be most valuable when targeting youth with light mental health problems as well as those with severe mental health problems who are not inclined to seek professional help.

Given the promise of serious games for mental health targeted at youth not (currently) in therapy, factors that increase motivation to engage with these games need to be addressed. It may be that promoting a serious game as a proven tool to enhance well-being could be effective. However, youth may not always be inclined to do something because it is ‘good for them’ and even if they do, their motivation may be lower than for a regular game. Another option may be a stealth approach which promotes mental health games as regular entertainment games.

The value of each approach will depend on how youth value addressing mental health concerns. On the one hand, youth who are actually seeking interventions may be more attentive to games with explicit mental health aims than to games using a stealth entertainment promotion. Games that are explicitly promoted for their mental health aims may be easier to locate (e.g., online game stores or platforms have health and well-being sections). Also, youth who are not actively seeking interventions but have mild symptoms may still be more attentive to information about mental health improvement as it is personally relevant for them.

On the other hand, the stealth approach may be very promising considering stigma and the reluctance of youth to seek treatment for mental health issues (Barney et al., 2006; Gulliver et al., 2010; Merikangas et al., 2011). Whereas explicitly promoted serious games may stand apart from ‘normal games,’ serious games may blend in with the available commercial games if they avoid explicit health messages both in their promotion and in the content of the game (e.g., not using psychoeducation; Buday, 2015). Additionally, youth who are (initially) resistant to the idea of treatment may avoid anything related to their mental health issues including a mental health game (e.g., out of embarrassment), while a stealth game is less likely to trigger resistance.

The current study examined the impact of explicit mental health and stealth entertainment promotion on game choice and game experience in young adults with elevated mental health symptoms. Hypotheses about the relative impact of these two approaches may be formed based on several theoretical models focused on media choice.

These models suggest that media selection can be based on a person’s needs, motivations (Katz et al., 1973), mood (Zillmann, 1988), mood deteriorating costs from media (Perse, 1998; Fahr and Bocking, 2009), long term benefits of media (Oliver, 2009), and a person’s desire to maintain their autonomy (Brehm, 1966; Burgoon et al., 2002). All of these models may indicate that explicit messaging would turn youth off to a mental health game. For example, reactance theory and the escape model suggest that health messages would drive youth to an alternative game, either because they perceive the message to threaten their choice freedom (Brehm, 1966; Hornik et al., 2008; Richards and Banas, 2015; Richards et al., 2017) or because they expect the game to induce negative emotions (e.g., an aim of stress management may imply confrontations with stress; Perse, 1998; Fahr and Bocking, 2009). However, many of these same theories may also be used to explain why youth may be attracted to explicit mental health games provided that youth have an interest in improving their well-being. For example, a wider definition of needs and costs from media suggests that youth would play an explicit mental health game to gain insight into personal issues and current negative emotions may be tolerated for long-term benefits (Oliver, 2009).

Next to media selection theories, the motivational theory of self-determination may predict youth’s responses to messaging depending on their intrinsic values. Intrinsic motivation (i.e., motivation stemming from the activity itself, e.g., the activity interests you) is theorized to be supported by three psychological needs being fulfilled: autonomy (experiencing the freedom to make your own decisions), competence (experiencing that you are able to be successful given your skills) and relatedness (experiencing a connection to others; Ryan and Deci, 2000). Self-determination theory is particularly interesting as need fulfillment and intrinsic motivation have been associated with better outcomes, including therapeutic outcomes (Ryan and Deci, 2000; Zuroff et al., 2007, 2017; Ryan et al., 2011). Moreover, Ryan et al. (2006) showed that experiencing autonomy and competence during gameplay is associated with game enjoyment, continued gameplay and better mood after gameplay. Initially, youth’s motivations (e.g., improving their mental health) and experienced need fulfillment (e.g., feeling autonomy is limited by the mental health message) may influence game choice. Thus, self-determination theory may be another theory that can explain youth’s game choice, although again it allows both hypothesis in favor of mental health messaging and in favor of entertainment messaging. Moreover, during gameplay intrinsic motivation and need fulfillment are elements of the game experience (i.e., experiencing low or high competence during gameplay) influenced by gameplay and potentially messaging. Therefore, intrinsic motivation and psychological needs may be vital when we try to understand the impact of messaging on game experience.

Thus, theoretically it is hard to predict how youth will react to serious games with an explicit or stealth promotional approach. Moreover, there is hardly any empirical evidence that can guide our hypotheses. In one closely related previous study, we examined how a mental health or entertainment trailer preceding a commercial video game influenced the experience of this game (Poppelaars et al., 2018). This study showed that even though all participants played the same game, game experience was influenced by the trailer that participants viewed. Although intrinsic motivation and changes in affect were equal for participants regardless of the trailer message, participants who saw the mental health message experienced less autonomy in the game. Also, participants who reported more depressive symptoms and saw the mental health message experienced less competence. Additionally, participants with more depressive symptoms increased their positive affect after gameplay. This suggested that gameplay may at least temporarily improve the depressed mood of those at elevated risk for a depressive disorder. In contrast to daily life, however, participants had no influence on which game they played.

Therefore, the current study was designed to replicate the main findings as well as expand the scope of the previous study in three ways. First, to better approximate real-world media decisions, the current study allowed participants to *choose* between games promoted with a mental health or entertainment message. Second, as serious games may be most valuable if they target youth with some level of mental health problems, we selected participants with elevated mental health symptoms. Finally, the current study assessed stress and anxiety symptoms, in addition to depressive symptoms, to broaden the scope of our understanding and inform future prevention efforts.

Our primary aim was to test how entertainment versus mental health messaging influenced the choice and experience of a video game in young adults with elevated mental health symptoms. Participants chose a game to play after viewing two trailers, one for each type of messaging. We were able to directly link messaging to differences in choice and experience as both trailers portrayed the same game, unbeknownst to the participants. Following gameplay, game experience was assessed. This allowed us to examine the effect of entertainment and mental health messaging on eight dependent variables in the whole sample. We studied the effect of trailer message on three indicators of game appeal: (1) game choice; (2) perceived attractiveness and (3) perceived fun of the game. Additionally, the effect of game choice was examined on five prominent aspects of game experience, that is: (4) gameplay duration; (5) intrinsic motivation; (6) autonomy; (7) competence and (8) change in affect. Based on the theoretical literature and lack of previous empirical evidence, we made no predictions about how the messaging would influence game choice, preference for the games (i.e., perceived attractiveness and fun of the games from the trailers), the duration of gameplay nor about changes in affect. However, based on previous results (Poppelaars et al., 2018), we expected gameplay after either trailer selection to result in similar levels of intrinsic motivation and competence and to improve affect equally. Furthermore, we hypothesized that participants would experience equal levels of autonomy because all participants selected the game they played.

The secondary aim of this study was to explore how severity and type of mental health symptoms influence game choice and experience. Thus, we explored if symptom severity or symptom type (i.e., depression, anxiety, and stress) moderated the effects of messaging on the same eight variables named above. Although there currently is no consensus on the relation between symptom severity and professional help-seeking, most evidence suggests that more severe symptoms are related to seeking more professional help (e.g., Oliver et al., 2005; Merikangas et al., 2011; Sawyer et al., 2012). Therefore, we hypothesized that youth with severe symptoms would select the mental health game more often. As the personal relevance of the mental health game is higher for those with severe mental health symptoms compared to those with less severe symptoms, we hypothesized that this choice is more intrinsically motivated and related to higher levels of autonomy. Furthermore, based on previous results (Poppelaars et al., 2018), we expected elevated depressive symptoms to predict a greater increase in affect and to predict less competence in participants who select the mental health game over the entertainment game. For anxiety, stress and the remaining dependent variables for depressive symptoms and symptom severity, no hypotheses were formulated.

## Materials and Methods

### Participants

In total 155 young adults (*M_age_* = 21.48, *SD_age_* = 3.36) participated in this study between March and November 2017. Participants were only included in the analyses if they were unaware of the study’s aims and the fact that both trailers reflected the same game (*n* = 129).

Participants included in the analyses were between 18 and 31 years old, with a mean age of 21.33 years (*SD* = 3.20). The majority of the participants was female 73.6%. Almost all participants were enrolled in or had completed higher education (91.5%), while some were enrolled in or had completed a pre-university track (7.0%). Two participants had completed unsegregated secondary education (1.6%). The majority of participants currently enrolled in education were enrolled in a social science track (76.9%).

All participants were selected based on having at least mildly elevated mental health symptoms on at least one subscale of the Depression Anxiety Stress Scale (DASS-21; Lovibond and Lovibond, 1995). Participants showed mildly elevated symptoms on one (41.9%), two (27.9%) or three (30.2%) subscales. At least a mildly elevated score on depression, anxiety or stress were shown by, respectively, 64.3%, 67.4%, and 56.6% of participants. Of the sample 31.8% had severe or extremely severe scores on at least one DASS-21 subscale.

Although participants indicated that they were moderately positive about video games in general (*M_liking_* = 4.50, *SD* = 1.75; on a 7-point scale with a higher score indicating a more positive attitude), 48.8% indicated not playing video games at all in an average week, with an additional 9.3% playing an hour or less per week. Almost a fifth of participants indicated playing video games more regularly (1–7 h a week; 18.6%) and almost a quarter of participants played more than 7 h a week (23.3%).

### Procedure

Participants were recruited for the study on a university and higher vocational education campus in the Netherlands through flyers and the university’s online research participation system. Young adults (*n* = 648), who provided informed consent, were invited to fill out a 15-min online screening questionnaire either voluntarily or for study credits. The online questionnaire was used to assess eligibility, as well as to gather information on demographics, video game behavior and additional questionnaires that are not part of the current study. The inclusion criteria were: (1) Being 18 years or older; (2) Having at least a mildly elevated score on the DASS-21; (3) Being unfamiliar with the game Monument Valley, meaning that the participant had not seen or heard anything about the game; and (4) Being willing and able to sign informed consent. Those who met the inclusion criteria (*n* = 264) were invited to participate in the lab experiment within 2 weeks from completing the screening questionnaire (range 1–20 days, *M* = 8.15, *SD* = 4.52). Of the 155 young adults participating in the lab experiment 9.0% participated between 2 and 3 weeks from screening, due to holiday periods and unavoidable delays (e.g., a participant being ill). Both the screening and experiment questionnaires were available in Dutch and English. For standardized questionnaires with no official translation, the Dutch translations of Poppelaars et al. (2018) were used (see article for further information).

During the lab experiment, participants were seated in a plain cubicle with a computer and a tablet. At the start of the experiment participants were given both verbal and written information on the study and gave informed consent. Participants were told that they would see two trailers of video games. The researcher requested the participants to choose the game they believed they would enjoy most, under the guise that the study’s topic was gameplay of a game that participants may have played at home. To further encourage choosing the most appealing game, participants were informed that they had a chance to win their chosen game.

Next, participants received instructions on using the tablet. They were told they were free to play the chosen game as long as they liked, but that it was important that they could evaluate the game. In fact, although there was no minimum time limit, participants were told to continue with the questionnaires 50 min from the start of the experiment (approximately 40 min of gameplay), to ensure they did not exceed the 60 min set for the experiment.

Once the researcher left the cubicle, participants filled out an assessment of affect. Next, participants were shown, in a random order, two trailers of the video game Monument Valley. One trailer portrayed the game as an entertainment game and one trailer portrayed the game as beneficial for players’ mental health. The trailers were designed to convince participants that two separate games were being promoted and the combination of trailer design and message was counterbalanced across participants. To achieve the latter, participants were randomized using a blocked randomization to receive trailer A including the entertainment message and trailer B including the mental health message or to see the same trailers with the messages interchanged. After viewing the trailers, participants were asked to select the game they would like most and were reminded that they could win that same game. After choosing a game, participants rated the attractiveness and fun of each game based on the trailer, before they were instructed to play Monument Valley 1 (Ustwo Games, 2014a).

Following gameplay, participants completed questionnaires on their affect, intrinsic motivation, autonomy and competence and questions about the manipulation check, trailer message and questionnaires not included in the current study. Participants were provided with study credits or a gift certificate worth €10. Once testing was completed in November 2017, debriefing was done through email and 10 participants were randomly selected to receive a reimbursement for purchasing Monument Valley 1 and 2 (Ustwo Games, 2014a, 2017). This study was approved by the ethical committee of the Faculty of Social Sciences at Radboud University (ECSW2017-3001-461).

### Monument Valley and Messaging

**FIGURE 1:**
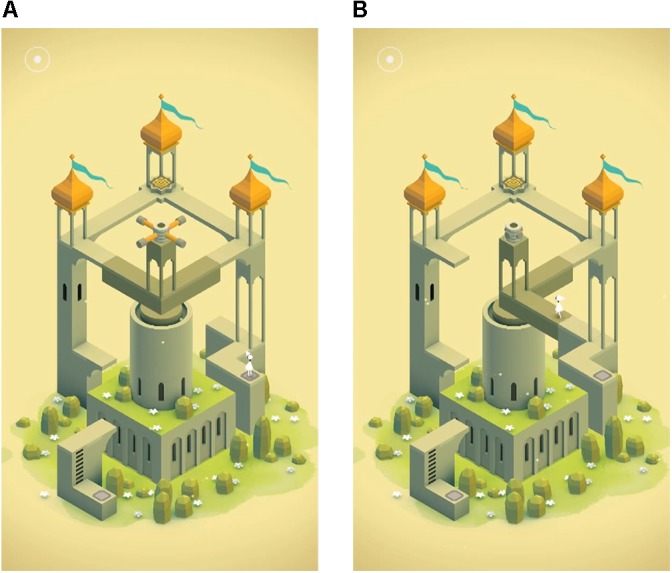
Screenshots from the video game Monument Valley. **(A)** A building before the dark khaki section is rotated. The player’s aim is to have the avatar in white reach the top of this building. **(B)** The same building after rotating the dark khaki section, allowing the player to find a new path upward. These images are reproduced from Monument Valley by Ustwo Games (2014a) with the permission of the copyright holder Ustwo Games.

The original Monument Valley game released in 2014 (Ustwo Games, 2014a) is an award winning commercial puzzle game with optical illusions inspired by the art of M. C. Escher and can be played on smartphones and tablets (**Figure 1**). This game was designed to create an optimal balance between difficulty and pleasure, as well as to allow all players to be able to complete all levels (Ustwo Games, 2014b) making it accessible for participants with various levels of gameplay experience. Although the game was not designed with a therapeutic aim, players may believe that it was when it is presented that way, because of the relaxed atmosphere and the way the game illustrates problem solving, an adaptive technique for coping with stress and negative emotions (i.e., the player finds solutions for the game’s challenges by literally looking at the challenge from several angles). Indeed, participant comments following gameplay indicated that the mental health claim was credible (e.g., one participant recommended the mental health game to other participants because ‘it will relax all stressed out students’).

**FIGURE 2:**
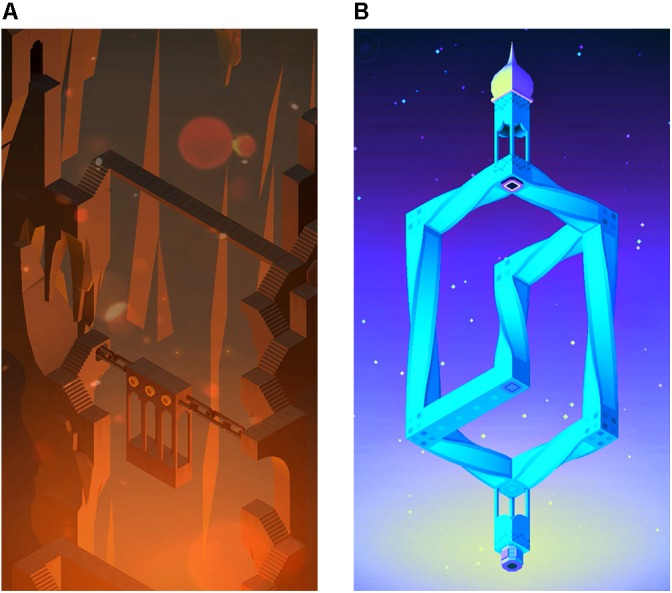
Screenshots from the two trailers of Monument Valley. **(A)** A screenshot from the detailed trailer. **(B)** A screenshot from the abstract trailer. These images have been adapted from Monument Valley by Ustwo Games (2014a) with the permission of the copyright holder Ustwo Games.

For this study, we created two distinguishable trailers of Monument Valley allowing us to attribute any differences in game choice or game experience to messaging while participants were unaware of this manipulation. Screenshots taken from the later levels and expansion levels of Monument Valley were used in the trailers to make it unlikely for participants to encounter these levels during the experiment. We differentiated the trailers on several aspects to create the impression that the trailers were portraying two separate games. Pilot studies were conducted to make sure that potential participants indeed believed that the two trailers advertised two different games. Information regarding pilot studies are available upon request. The trailers, which we will refer to as the *detailed* and the *abstract* trailer, were both approximately 1-min long and differed on the following aspects, respectively: (1) Showing game challenges that had a more *detailed* environment vs. game challenges that were *abstract* buildings floating in space (**Figure 2**); (2) Faster vs. slower music; (3) A warmer vs. a cooler color palette; (4) AR BONNIE vs. Gloucester MT font for the trailer text; and (5) An editing style focused on slowly moving across the pictures vs. zooming in or out of the pictures.

During the experiment, the messaging in each trailer design was counterbalanced and trailers were shown in a random order. This allowed us to assess the effects of messaging while controlling for trailer design. Approximately half of the participants saw the detailed trailer as the mental health trailer (*n* = 66) and the abstract trailer as the entertainment trailer, while the other half (*n* = 63) saw the abstract trailer as the mental health trailer and the detailed trailer as the entertainment trailer. Both messages consisted of five short phrases. For both messages the first sentence introduced the game as appealing, as one would expect from a promotional trailer. The next four phrases focused on the mental health or the entertainment message (**Table 1**).

**Table 1:** Messages included in the mental health and entertainment trailers.

Mental health	Entertainment
Perfect for a single marathon playthrough	A game you must play
Learn to manage stress more efficiently	Think outside the box to solve intricate puzzles
Therapeutic insights for emotional mastery	9/10 Polygon 5/5 Touch Arcade
Both challenging and relaxing	Almost impossibly gorgeous
Recommended by games for mental health	IPad game of the year

### Instruments

#### Mental Health Symptoms

The DASS-21 (Lovibond and Lovibond, 1995) was used both in the original English version as well as in the Dutch translation by De Beurs (2010). All 21-items were scored on a 4-point scale (*Did not apply to me at all* = 0, *Applied to me to some degree, or some of the time* = 1, *Applied to me to a considerable degree, or a good part of the time* = 2, *Applied to me very much, or most of the time* = 3) with seven items for each of the subscales: depressive symptoms (e.g., ‘I felt that I had nothing to look forward to.’; α = 0.83), anxiety symptoms (e.g., ‘I felt scared without any good reason.’; α = 0.70) and stress symptoms (e.g., ‘I found it difficult to relax.’; α = 0.79). Participants rated to what degree each statement applied to them over the past week. The summed score for each subscale indicated mild symptoms at or above a score of 5 for depression, 4 for anxiety, and 8 for stress. Thus, when we refer to elevated symptoms, we are referring to symptoms at or above the mild cut-off. Additionally, in the analyses all participants who scored at or above the severe cutoff (11 for depression, 8 for anxiety, and 13 for stress) for one or more of the subscales were compared to participants who had no symptoms within the severe or extremely severe range. As symptoms of the different subscales have different cutoffs (e.g., a score of 8 can indicate mild stress or severe anxiety) a continuous sum score cannot be used to indicate overall mental health. Thus, when referring to severe symptoms, we are referring to a score at or above the severe cut-off on one or more DASS-21 subscales.

#### Affect

The Self-Assessment Manikin (SAM; Bradley and Lang, 1994) for affect was used to asses current affect before and after gameplay. Participants were asked to indicate, on a 5-point manikin based scale, the manikin that reflected how they felt at that moment and were given a list of adjectives to indicate the extreme negative (e.g., *unhappy, annoyed, bored* = 1) and extreme positive (e.g., *happy, satisfied, hopeful* = 5) points of the scale. Each manikin was a simple line drawing of a person with an emotional facial expression, with the neutral facial expression in the middle of the scale (a score of 3).

#### Game Choice and Trailer Preference

After viewing the trailers, participants chose one of the games, referred to as game A and game B and accompanied by screenshots to ensure that participants could correctly identify each game. Although all participants played Monument Valley, the analyses were done from the perspective of the participant and focus on their choice for a game promoted with mental health or entertainment messaging. Directly after game selection, participants rated both games based on the trailers on attractiveness and fun using two separate 10-point scales (1–10), with higher scores indicating more perceived attractiveness and perceived fun, respectively.

#### Gameplay Duration

Gameplay duration was measured using two methods. First, the online questionnaire page that was open while participants played Monument Valley contained an invisible timer. Second, the game tablets were equipped with the program Funamo Parental Control (Funamo; Funamo Inc., 2016), which recorded how long Monument Valley was open for each participant.

Both measures were used to create a gameplay duration measure, as some participants did not close the game when they continued with the questionnaire (resulting in an incorrect gameplay duration in Funamo) and others continued to the next page in the questionnaire before finishing gameplay (resulting in an incorrect gameplay duration on the questionnaire timer). As Funamo directly records how long the game is opened it was the preferred measure. For participants who had an incorrect gameplay duration in Funamo (*n* = 9), we used the gameplay duration from the questionnaire corrected for the average time it took to open and close the game as well as to read the instruction on the questionnaire page. For one participant, no gameplay duration could be calculated as both the Funamo and questionnaire measures were incorrect.

Additionally, it was decided that a standard gameplay duration would be given to all participants who were stopped during gameplay to complete the questionnaire (*n* = 25) and participants who exceeded that standard duration without being stopped (*n* = 2). This was done because the maximum amount of time participants could play during the experiment was constrained by the duration of the experiment explanation and the time it took participants to fill out all questions prior to gameplay. Thus, the gameplay duration for all these participants was set at the mean gameplay duration correctly recorded for participants who were stopped, that is 40.38 min.

#### Intrinsic Motivation

Intrinsic motivation was measured with the interest/enjoyment subscale from the Intrinsic Motivation Inventory (Ryan, 1982; McAuley et al., 1989). Participants responded to seven statements (e.g., ‘This game was fun to do.’) about their experience with Monument Valley using a 7-point scale (*Not at all true* = 1, *Somewhat true* = 4, *Very true* = 7). Two items needed to be recoded to create a mean score where higher scores indicated more intrinsic motivation (α = 0.89).

#### Autonomy and Competence

Using the Player Experience of Need Satisfaction questionnaire (PENS; Ryan et al., 2006; Immersyve, 2007), the psychological needs autonomy and competence were measured. Both needs were assessed with three items, for example ‘The game lets you do interesting things.’ for autonomy (α = 0.78) and for example ‘I feel very capable and effective when playing.’ for competence (α = 0.83). Items were rated on a 7-point scale (*Strongly disagree* = 1, *Strongly agree* = 7) and a mean was calculated for each subscale with higher scores indicating a stronger experience of autonomy and competence, respectively. The psychological need for relatedness was not measured as Monument Valley provides no opportunity to interact with other players.

#### Manipulation Check

At the end of the experiment participants were asked (1) what they believed the study’s aim was, (2) which game they would recommend to the next participant and why (i.e., *Game A, because …, Game B, because …, or I do not have a preference, because …*, with the last option prompting some participants to indicate that they believed there was only one game) and (3) to explain the difference between the messaging of the two trailers, if they had noticed a difference. The answers to these questions were checked for (1) awareness of the study’s manipulation using one game rather than two games; (2) awareness of the study’s aim to relate the mental health message in one of the trailers to game choice and (3) awareness of the study’s aim to relate players mental health symptoms to game choice. Participants who were aware of at least one, were excluded from the analyses (*n* = 26).

#### Trailer Message Awareness

After the manipulation check, participants answered two last questions to identify if they had noticed the mental health message. The first question was ‘Did you notice that the message of one of the two trailers was primarily focused on game enjoyment, while the other trailer contained the message that it could help people who feel stressed or have some mental health difficulties?’ (*Yes* or *No*). Finally, participants selected a screenshot from one of the trailers to answer the question ‘Which of the two trailers do you believe contained the message that this video game can help people who feel stressed or have some mental health difficulties?’

### Statistical Analyses

All analyses were done in version 25 of SPSS (IBM Corp, 2017). First, randomization was checked by comparing the two conditions on several descriptive variables, mental health symptoms and awareness of messaging using *t*-tests and Chi-square. Additionally, a Chi-square test was used to compare the selection of the trailer designs and dependent *t*-tests were used to test if perceived attractiveness and perceived fun differed per trailer design. Also, *t*-tests were done to test if the trailer design of the chosen game was related to gameplay duration, intrinsic motivation, autonomy, competence, affect. Additionally, a Repeated Measures ANOVA (RM-ANOVA) was used to relate changes in affect to the trailer design of the chosen game.

Turning to the main research aim, a logistic regression was performed predicting game choice using trailer design as a predictor to understand how the effects of trailer design and trailer message interact. Furthermore, *t*-tests were used to test if game choice was related to gameplay duration, intrinsic motivation, autonomy, competence, and affect before and after gameplay. Moreover, for perceived attractiveness and fun RM-ANOVAs were used to compare the scores for the mental health and the entertainment trailer provided by each participant. Another RM-ANOVA was used to relate changes in affect to game choice.

Finally, these analyses were repeated to address the second research aim to distinguish effects of mental health symptom severity and type of mental health symptoms. First, in logistic regressions game choice was predicted using trailer design, symptoms and the interaction between symptoms and trailer design. Next, one-way ANOVAs were used to relate symptoms and the interaction between symptoms and game choice to gameplay duration, intrinsic motivation, autonomy, competence, and affect before and after gameplay. Again, RM-ANOVAs were used for attractiveness, fun and affect. Thus, the analyses were repeated comparing those with and without severe symptoms, those with or without elevated depressive symptoms, those with or without elevated anxiety symptoms and those with or without elevated stress symptoms. For all significant interaction effects *post hoc* analyses were performed with a Bonferroni correction.

## Results

### Descriptive Statistics

The descriptive statistics for the entire sample and per condition are provided in **Table 2**. Randomization was successful as there were no differences between conditions on age, gender, birth country, general video game liking, weekly hours of video gameplay, depressive symptoms, anxiety symptoms, stress symptoms or severe symptoms. Participants played Monument Valley for a mean of 28.48 min (*SD* = 8.85), with a range of 12.77–40.38 min.

**Table 2:** Descriptives (means and standard deviations or percentages) for the total sample and per condition including chi-square tests and *t*-tests comparing conditions.

	Total (*SD*)		Detailed trailer with mental health (*SD*)		Abstract trailer with mental health (*SD*)		*X^2^/t*	*df*	*p*
**Age**	21.33	(3.20)	21.56	(3.06)	21.10	(3.35)	0.83	127	0.41
**Gender**							0.06	1	0.81
Female	73.6%		72.7%		74.6%				
Male	26.4%		27.3%		25.4%				
**Birth country**							1.77	2	0.41
Dutch	49.6%		53.0%		46.0%				
German	29.5%		24.2%		34.9%				
Other	20.9%		22.7%		19.0%				
**Video game liking**	4.50	(1.75)	4.55	(1.85)	4.46	(1.64)	0.28	127	0.78
**Weekly video gameplay**	4.25	(6.77)	4.83	(7.52)	3.63	(5.87)	1.01	122.20	0.31
**Depressive symptoms**	6.02	(3.92)	6.30	(4.16)	5.71	(3.67)	0.85	127	0.40
**Anxiety symptoms**	5.12	(3.41)	5.52	(3.75)	4.70	(3.00)	1.36	127	0.18
**Stress symptoms**	8.24	(3.85)	8.35	(4.04)	8.13	(3.67)	0.33	127	0.75
**Severe symptoms**							<0.01	1	0.99
Yes	31.8%		31.8%		31.7%				
No	68.2%		68.2%		68.3%				
**Elevated depressive symptoms**							0.29	1	0.59
Yes	64.3%		62.1%		66.7%				
No	35.7%		37.9%		33.3%				
**Elevated anxiety symptoms**							0.88	1	0.35
Yes	67.4%		71.2%		63.5%				
No	32.6%		28.8%		36.5%				
**Elevated stress symptoms**							0.70	1	0.40
Yes	56.6%		53.0%		60.3%				
No	43.4%		47.0%		39.7%				
**Messaging identified**							0.32	1	0.57
Yes	82.2%		80.3%		84.1%				
No	17.8%		19.7%		15.9%				

Moreover, we tested if the mental health message was clear in both trailer designs. Of the whole sample 88.4% indicated that they noticed that one of the trailers contained a mental health message and 82.2% was able to correctly identify this trailer. There was no difference between conditions in correctly identifying the trailer containing the mental health message (**Table 2**). Furthermore, there was no difference in awareness of the mental health message between those who decided to play the mental health game versus those who decided to play the entertainment game (81.8% vs. 82.7% correctly identified the mental health trailer, respectively; *X^2^* (1, *n* = 129) = 0.02, *p* = 0.90).

### Trailer Design

Before testing the effect of the trailer message, we tested if trailer designs were selected equally and if trailer design affected preference (**Table 3**). Participants were significantly more likely to select the detailed trailer than the abstract trailer. Similarly, participants perceived the game in the detailed trailer as more attractive and more fun than the abstract trailer. Therefore, trailer design was controlled for in all further analyses on game choice, perceived attractiveness and perceived fun.

**Table 3:** Descriptives (means and standard deviations or percentages) for the detailed and abstract trailers including chi-square and *t*-tests comparing trailers.

	Detailed trailer (*SD*)		Abstract trailer (*SD*)		*X*^2^*/t*	*df*	*p*
Game choice	69.8%		30.2%		20.16	1	<0.001
Attractive	6.50	(1.60)	5.53	(1.83)	-6.07	128	<0.001
Fun	6.25	(1.62)	5.47	(1.81)	-5.25	128	<0.001
Affect before	3.73	(0.73)	3.67	(0.81)	-0.46	127	0.65
Affect after	3.89	(0.67)	3.87	(0.67)	-0.14	124	0.89
Duration play	28.32	(8.91)	28.86	(8.82)	0.32	126	0.75
Motivation	5.25	(1.04)	5.38	(0.97)	0.67	127	0.51
Autonomy	4.75	(1.20)	4.95	(1.03)	0.89	127	0.37
Competence	4.98	(1.11)	5.10	(1.19)	0.56	127	0.58

Next, we tested whether the trailer design of the chosen game was related to game experience. We found that affect increased over time with a small effect size [*F*(1, 124) = 6.45, *p* < 0.05, ${\text{η}}_{\text{p}}^{2}$ = 0.05], however, the trailer design of the chosen game was not related to change of affect over time [*F*(1, 124) = 0.01, *p* = 0.92, ${\text{η}}_{\text{p}}^{2}$ < 0.01]. In addition, results show that trailer design was not related to affect before gameplay, affect after gameplay, duration of gameplay or the experience of intrinsic motivation, autonomy or competence (**Table 3**). Thus, participants who played Monument Valley after selecting the detailed trailer did not play longer and did not experience more intrinsic motivation, autonomy or competence than participants who played the game after selecting the abstract trailer.

### Main Analyses

**FIGURE 3:**
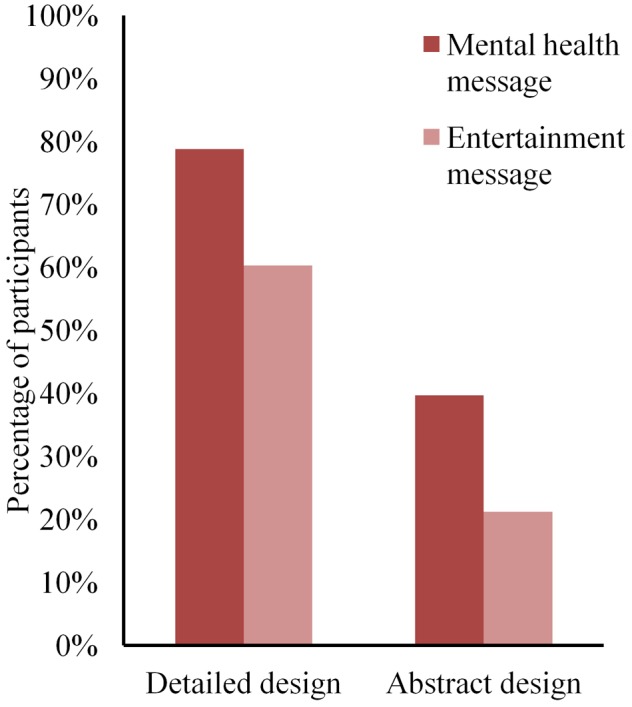
The percentage of game selection. Participants were given the choice between (1) the detailed trailer with the mental health message (78.8%) and the abstract trailer with the entertainment message (21.2%) or (2) the detailed trailer with the entertainment message (60.3%) and the abstract trailer with the mental health message (39.7%). Despite the detailed design being preferred over the abstract design, both designs were chosen more often when presented with the mental health message.

To address the first research aim, we first compared the effects of trailer message on game choice and preference. A logistic regression predicting game choice using the trailer design as a predictor showed that the odds of choosing the mental health game were 3.71 times higher than the odds of choosing the entertainment game [*X^2^* (1, *n* = 129) = 18.99, *p* < 0.001]. However, the odds of choosing the mental health message when it was portrayed in the detailed trailer were 5.65 times higher than the odds of choosing the mental health message when it was portrayed in the abstract trailer [*X^2^* (1, *n* = 129) = 19.09, *p* < 0.001]. The complete model was able to correctly predict 69.8% of the game choices [*X^2^* (1, *n* = 129) = 21.11, *p* < 0.001]. **Figure 3** shows how the four combinations of trailer design and trailer message were chosen at different rates by the participants. Specifically, the figure shows both a favoring of the detailed trailer and the trailer with the mental health message.

**FIGURE 4:**
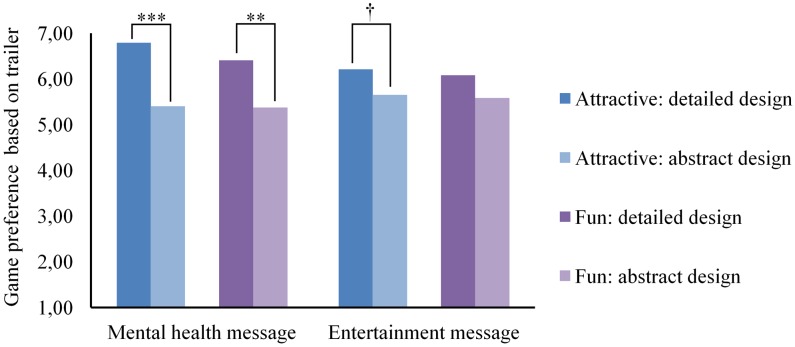
Perceived attractiveness and fun of the game for each design and message combination. Significant effects are indicated as follows: ^†^*p* < 0.10, ^∗^*p* < 0.05, ^∗∗^*p* < 0.01, and ^∗∗∗^*p* < 0.001.

Although mental health messaging made it more likely for participants to choose a game, participants did not perceive the game promoted with the mental health message as more attractive or more fun than the game promoted with the entertainment message (**Table 4**). However, there was a significant interaction for perceived attractiveness of trailer message × trailer design [*F*(1, 127) = 36.60, *p* < 0.001, ${\text{η}}_{\text{p}}^{2}$ = 0.22], indicating that there was a significant difference between the trailer designs in perceived attractiveness when they contained the mental health message [*M_abstract_* = 5.40, *SE_abstract_* = 0.21; *M_detailed_* = 6.79, *SE_detailed_* = 0.20; *F*(1, 127) = 22.89, *p* < 0.001, ${\text{η}}_{\text{p}}^{2}$ = 0.15], but not when the trailer designs contained the entertainment message [*M_abstract_* = 5.65, *SE_abstract_* = 0.22; *M_detailed_* = 6.21, *SE_detailed_* = 0.22; *F*(1, 127) = 3.16, *p* = 0.08, ${\text{η}}_{\text{p}}^{2}$ = 0.02]. Similarly, there was a significant interaction for perceived fun of trailer message × trailer design [*F*(1, 127) = 27.23, *p* < 0.001, ${\text{η}}_{\text{p}}^{2}$ = 0.18], indicating that there was a significant difference between the trailer designs in perceived fun when they contained the mental health message [*M_abstract_* = 5.37, *SE_abstract_* = 0.21; *M_detailed_* = 6.41, *SE_detailed_* = 0.21; *F*(1, 127) = 12.51, *p* < 0.001, ${\text{η}}_{\text{p}}^{2}$ = 0.09] and not when they contained the entertainment message [*M_abstract_* = 5.58, *SE_abstract_* = 0.22; *M_detailed_* = 6.08, *SE_detailed_* = 0.22; *F*(1, 127) = 2.64, *p* = 0.11, ${\text{η}}_{\text{p}}^{2}$ = 0.02]. Thus, participants believed the game to be significantly more fun and attractive if the mental health message was included in the detailed trailer than if they received the mental health message in the abstract trailer. However, when trailers contained the entertainment message the abstract and the detailed trailer were perceived as equally fun and attractive (**Figure 4**).

**Table 4:** Descriptives (means and standard deviations or percentages) for the mental health and entertainment trailers including tests comparing trailers: chi-square, RM-ANOVA and *t*-tests.

	Mental health trailer (*SD*)		Entertainment trailer (*SD*)		*X*^2^*/t/F*	*df*	*p*
Game choice	59.7%		40.3%		4.85	1	<0.05
Attractive	6.11	(1.79)	5.92	(1.79)	1.03	1, 127	0.31
Fun	5.90	(1.75)	5.82	(1.77)	0.16	1, 127	0.69
Affect before	3.78	(0.77)	3.62	(0.72)	1.22	127	0.23
Affect after	3.95	(0.57)	3.79	(0.78)	1.25	88.28	0.16
Duration play	28.74	(8.87)	28.10	(8.90)	0.40	126	0.69
Motivation	5.31	(1.03)	5.26	(1.01)	0.27	127	0.79
Autonomy	4.81	(1.15)	4.81	(1.16)	-0.02	127	0.98
Competence	4.99	(1.16)	5.06	(1.10)	-0.38	127	0.71

*The Chi-square test for game choice was not controlled for trailer design*.

Furthermore, we tested if game choice predicted game experience (**Table 4**). *T*-tests showed no differences in gameplay duration, intrinsic motivation, autonomy and competence. Thus, playing a game promoted for entertainment or mental health did not change the duration of gameplay, nor how much intrinsic motivation, autonomy and competence players experienced.

Next, we tested change in affect. As shown previously there was a significant improvement in affect over time [*F*(1, 124) = 7.18, *p* < 0.01, ${\text{η}}_{\text{p}}^{2}$ = 0.06], however, there was no effect of game choice on the change in affect [*F*(1, 124) < 0.01, *p* = 0.98, ${\text{η}}_{\text{p}}^{2}$ < 0.01]. This indicates that participants experienced more positive affect after gameplay regardless of playing a game promoted for entertainment or mental health.

### Exploratory Analyses

In order to address the second research aim, we explored the effects of mental health symptom severity and type on all dependent variables. For all groups, we first tried to predict game choice with a logistic regression using symptoms and the interaction between symptoms and trailer design. Next, the remaining dependent variables were predicted using symptoms and the interaction between symptoms and game choice as predictors. All descriptives and statistics can be found in **Table 5**, with the exception of the three-way interactions for fun and attractiveness and the RM-ANOVAs for change in affect.

**Table 5:** Descriptives (means and standard deviations) and ANOVA results: main effect of symptoms and interaction with messaging.

		Total				Mental health		Entertainment			
		Non-elevated *M (SD)*	Elevated *M (SD)*	*F*	${\text{η}}_{\text{p}}^{2}$	Non-elevated *M (SD)*	Elevated *M (SD)*	Non-elevated *M (SD)*	Elevated *M (SD)*	*F*	${\text{η}}_{\text{p}}^{2}$
**Symptom severity**											
	Attractive	5.98 (1.54)	6.10 (1.28)	0.21^a^	<0.01	6.17 (1.84)	5.98 (1.68)	5.78 (1.86)	6.22 (1.60)	3.39^a†^	0.03
	Fun	5.78 (1.56)	6.04 (1.35)	0.82^a^	<0.01	5.89 (1.79)	5.93 (1.68)	5.67 (1.84)	6.15 (1.59)	1.99^a^	0.02
	Affect before	3.83 (0.71)	3.45 (0.78)	**6.66^c∗^**	0.05	3.92 (0.67)	3.48 (0.87)	3.70 (0.74)	3.40 (0.63)	0.22c	<0.01
	Affect after	3.92 (0.71)	3.80 (0.56)	0.44^c^	<0.01	4.04 (0.54)	3.76 (0.60)	3.76 (0.86)	3.87 (0.52)	2.28c	0.02
	Duration play	28.31 (9.27)	28.84 (8.01)	0.02^b^	<0.01	28.27 (9.46)	29.66 (7.72)	28.36 (9.14)	27.44 (8.56)	0.44b	<0.01
	Motivation	5.19 (1.07)	5.52 (0.87)	2.97^a†^	0.02	5.21 (1.06)	5.50 (0.96)	5.15 (1.09)	5.54 (0.70)	0.08^a^	<0.01
	Autonomy	4.63 (1.21)	5.20 (0.89)	**4.80^a∗^**	0.04	4.50 (1.19)	5.41 (0.79)	4.80 (1.24)	4.84 (0.97)	**3.97^a∗^**	0.03
	Competence	4.87 (1.20)	5.34 (0.91)	**5.13^a∗^**	0.04	4.84 (1.24)	5.27 (0.95)	4.90 (1.16)	5.47 (0.85)	0.10^a^	<0.01
**Depressive symptoms**											
	Attractive	6.12 (1.63)	5.96 (1.36)	0.23^a^	<0.01	6.22 (1.92)	6.05 (1.72)	6.02 (1.97)	5.87 (1.69)	0.03^a^	<0.01
	Fun	5.77 (1.60)	5.91 (1.45)	0.31^a^	<0.01	5.85 (1.83)	5.93 (1.72)	5.70 (1.91)	5.89 (1.70)	0.05^a^	<0.01
	Affect before	4.02 (0.61)	3.52 (0.76)	**16.43^c∗∗∗^**	0.12	4.00 (0.71)	3.65 (0.78)	4.05 (0.50)	3.32 (0.70)	2.04c	0.02
	Affect after	4.04 (0.60)	3.79 (0.69)	**4.47^c∗^**	0.04	4.16 (0.47)	3.84 (0.59)	3.90 (0.70)	3.71 (0.82)	0.27c	<0.01
	Duration play	27.00 (9.10)	29.31 (8.66)	1.23^b^	0.01	25.42 (8.59)	30.37 (8.63)	28.88 (9.53)	27.56 (8.57)	3.66^b†^	0.03
	Motivation	5.11 (1.16)	5.39 (0.92)	2.54^a^	0.02	5.19 (1.20)	5.37 (0.95)	5.01 (1.14)	5.43 (0.88)	0.42^a^	<0.01
	Autonomy	4.63 (1.28)	4.91 (1.07)	1.44^a^	0.01	4.52 (1.36)	4.95 (1.02)	4.76 (1.19)	4.85 (1.16)	0.63^a^	<0.01
	Competence	4.93 (1.17)	5.06 (1.12)	0.30^a^	<0.01	4.81 (1.33)	5.07 (1.08)	5.08 (0.95)	5.05 (1.21)	0.44^a^	<0.01
**Anxiety symptoms**											
	Attractive	6.35 (1.18)	5.86 (1.56)	3.73^a†^	0.03	6.48 (1.45)	5.93 (1.91)	6.21 (1.76)	5.78 (1.79)	0.70^a^	0.01
	Fun	6.30 (1.18)	5.65 (1.59)	**6.09^a∗^**	0.05	6.36 (1.45)	5.68 (1.85)	6.24 (1.53)	5.62 (1.85)	0.38^a^	<0.01
	Affect before	3.54 (0.81)	3.79 (0.71)	2.93^c†^	0.02	3.58 (0.83)	3.86 (0.73)	3.47 (0.80)	3.69 (0.68)	0.05c	<0.01
	Affect after	3.80 (0.68)	3.92 (0.66)	0.47^c^	<0.01	3.79 (0.59)	4.02 (0.55)	3.82 (0.81)	3.77 (0.77)	1.20c	0.01
	Duration play	30.87 (8.48)	27.31 (8.84)	3.60^b†^	0.03	32.22 (7.60)	27.04 (9.02)	28.88 (9.53)	27.71 (8.70)	1.44b	0.01
	Motivation	5.44 (1.03)	5.22 (1.01)	1.12^a^	0.01	5.51 (0.92)	5.21 (1.08)	5.34 (1.19)	5.22 (0.92)	0.24^a^	<0.01
	Autonomy	4.86 (1.15)	4.79 (1.15)	0.07^a^	<0.01	4.88 (1.14)	4.78 (1.16)	4.82 (1.21)	4.81 (1.16)	0.04^a^	<0.01
	Competence	5.08 (1.25)	4.99 (1.08)	0.15^a^	0.01	5.07 (1.34)	4.95 (1.07)	5.10 (1.13)	5.05 (1.11)	0.02^a^	<0.01
**Stress symptoms**											
	Attractive	5.98 (1.37)	6.04 (1.53)	0.13^a^	<0.01	6.21 (1.68)	6.03 (1.87)	5.75 (1.70)	6.05 (1.86)	1.16^a^	0.01
	Fun	5.74 (1.56)	5.95 (1.45)	0.77^a^	0.01	5.91 (1.75)	5.89 (1.76)	5.57 (1.80)	6.01 (1.74)	1.39^a^	0.01
	Affect before	3.72 (0.71)	3.69 (0.78)	0.04^c^	<0.01	3.78 (0.71)	3.76 (0.82)	3.64 (0.73)	3.60 (0.72)	<0.01c	<0.01
	Affect after	3.91 (0.65)	3.86 (0.68)	0.08^c^	<0.01	4.00 (0.51)	3.90 (0.62)	3.77 (0.81)	3.80 (0.76)	0.25c	<0.01
	Duration play	28.65 (9.31)	28.36 (8.56)	<0.01^b^	<0.01	29.54 (9.23)	28.13 (8.65)	27.30 (9.50)	28.68 (8.55)	0.74b	0.01
	Motivation	5.20 (1.10)	5.36 (0.95)	0.94^a^	0.01	5.27 (1.20)	5.34 (0.89)	5.09 (0.93)	5.39 (1.05)	0.38^a^	<0.01
	Autonomy	4.66 (1.30)	4.93 (1.02)	1.49^a^	0.01	4.64 (1.31)	4.95 (0.99)	4.70 (1.30)	4.90 (1.06)	0.06^a^	<0.01
	Competence	5.11 (1.11)	4.95 (1.15)	0.79^a^	0.01	5.02 (1.27)	4.96 (1.08)	5.24 (0.82)	4.93 (1.27)	0.37^a^	<0.01

*^a^ df = (1,125), ^b^ df = (1,124), ^c^ df = (1,122), ^†^p < 0.10, ^∗^p < 0.05, ^∗∗^p < 0.01, ^∗∗∗^p < 0.001, significant effects are printed in bold*.

#### Symptom Severity

For game choice, symptom severity was not found to be related either directly [*X^2^* (1, *n* = 129) = 0.86, *p* = 0.35] or in interaction with trailer design [*X^2^* (1, *n* = 129) = 0.51, *p* = 0.48]. Thus, participants without severe symptoms were equally likely to select the mental health game (58.0%) as participants with severe symptoms (63.4%).

However, further analyses did show effects of symptom severity on competence and autonomy. A direct effect was found for competence, demonstrating that participants with severe symptoms experienced more competence in the game than participants without severe symptoms regardless of game choice. Additionally, both a direct and an interaction effect was found for autonomy. Together these effects show that for those who selected the entertainment game, no difference was found on autonomy for participants with or without severe symptoms [*F*(1, 125) = 0.02, *p* = 0.90, ${\text{η}}_{\text{p}}^{2}$ < 0.01]. However, when participants selected the mental health game, participants with severe symptoms experienced more autonomy in the game than participants without severe symptoms [*F*(1, 125) = 11.43, *p* < 0.001, ${\text{η}}_{\text{p}}^{2}$ = 0.08].

Moreover, we observed that affect was lower prior to, but not after, gameplay in participants with severe symptoms than in participants without severe symptoms. Further analyses showed an interaction of time × symptom severity [*F*(1, 122) = 4.16, *p* < 0.05, ${\text{η}}_{\text{p}}^{2}$ = 0.03], but no three-way interaction: time × game choice × symptom severity [*F*(1, 122) = 0.83, *p* = 0.36, ${\text{η}}_{\text{p}}^{2}$ = 0.01]. *Post hoc* tests suggested that there was an increase in affect for participants with severe symptoms [*F*(1, 122) = 10.30, *p* < 0.01, ${\text{η}}_{\text{p}}^{2}$ = 0.08], but affect did not change for participants without severe symptoms [*F*(1, 122) = 1.29, *p* = 0.26, ${\text{η}}_{\text{p}}^{2}$ = 0.01]. In sum, participants with more severe symptoms experienced less positive affect prior to gameplay, but showed an increase in positive affect, resulting in equal positive affect for participants with more and less severe symptoms after gameplay. Symptom severity had no further effects on the dependent variables.

#### Depressive Symptoms

Elevated depressive symptoms did not influence game choice either directly [*X^2^* (1, *n* = 129) = 2.69, *p* = 0.10] or in interaction with trailer design [*X^2^* (1, *n* = 129) = 1.22, *p* = 0.27], which indicates that there was no difference in the odds for participants with and without elevated depressive symptoms to select the mental health game (62.7% and 54.3%, respectively).

Also, depressive symptoms did not predict dependent variables except for affect. Elevated depressive symptoms were related to less positive affect both before and after gameplay. Further analyses showed that there was an interaction for time × elevated depressive symptoms on affect [*F*(1, 122) = 4.40, *p* < 0.05, ${\text{η}}_{\text{p}}^{2}$ = 0.04], indicating that affect became more positive for participants with elevated depressive symptoms [*F*(1, 122) = 12.45, *p* < 0.001, ${\text{η}}_{\text{p}}^{2}$ = 0.09], while affect did not change for participants without elevated depressive symptoms [*F*(1, 122) = 0.01, *p* = 0.94, ${\text{η}}_{\text{p}}^{2}$ < 0.01]. There was no three-way interaction: time × game choice × elevated depressive symptoms [*F*(1, 122) = 3.68, *p* = 0.06, ${\text{η}}_{\text{p}}^{2}$ = 0.03]. This effect replicates what we previously saw for severe symptoms.

#### Anxiety Symptoms

We found no direct effect of anxiety symptoms [*X^2^* (1, *n* = 129) = 0.46, *p* = 0.50], nor an interaction effect of anxiety symptoms × trailer design on game choice [*X^2^* (1, *n* = 129) = 0.34, *p* = 0.56]. Thus, anxiety symptoms did not predict how often the mental health trailer was selected (non-elevated symptoms: 59.5%; elevated symptoms: 59.8%).

Moreover, no main or interaction effects of anxiety symptoms with game choice were found on gameplay duration, intrinsic motivation, autonomy, competence, attractiveness, or affect before and after gameplay. However, there was a significant effect of anxiety symptoms on perceived fun of the trailers, showing that participants with elevated anxiety symptoms rated the games as less fun than participants without these symptoms based on the trailers. Further analyses did not show an interaction of time × anxiety symptoms [*F*(1, 122) = 1.29, *p* = 0.26, ${\text{η}}_{\text{p}}^{2}$ = 0.01] or a three-way interaction of time × game choice × anxiety symptoms [*F*(1, 122) = 0.62, *p* = 0.43, ${\text{η}}_{\text{p}}^{2}$ = 0.01] on affect. Thus, anxiety symptoms did not influence the change in affect after gameplay.

#### Stress Symptoms

Finally, elevated stress symptoms did not influence the odds of selecting the mental health or the entertainment trailer directly [*X^2^* (1, *n* = 129) = 2.07, *p* = 0.15], nor in interaction with trailer design [*X^2^* (1, *n* = 129) = 3.25, *p* = 0.07]. Therefore, it appears participants with elevated stress symptoms were no more likely to select the mental health trailer than participants without elevated stress symptoms (58.9% and 60.7%, respectively).

Furthermore, no significant direct or interaction effects of stress symptoms were found on gameplay duration, intrinsic motivation, autonomy, competence, perceived attractiveness, fun, or affect before and after gameplay. Moreover, there was no interaction of time × stress symptoms [*F*(1, 122) < 0.01, *p* = 0.96, ${\text{η}}_{\text{p}}^{2}$ < 0.01] nor a three-way interaction of time × game choice × stress symptoms [*F*(1, 122) = 0.28, *p* = 0.60, ${\text{η}}_{\text{p}}^{2}$ < 0.01] for affect. Thus, participants with elevated stress symptoms did not react differently to the game than participants without elevated stress symptoms.

#### Affect

As previous analyses showed that affect only improves in participants with severe symptoms and participants with elevated depressive symptoms, but not in other participants, we examined if severe depressive symptoms are driving the effect. Therefore, we performed RM-ANOVAs for change in affect using only severe symptoms within each type of mental health symptoms as a predictor. These analyses showed that only those with severe depressive symptoms increased in affect [*F*(1, 122) = 5.65, *p* < 0.05, ${\text{η}}_{\text{p}}^{2}$ = 0.04], in contrast to those with severe anxiety [*F*(1, 122) = 0.82, *p* = 0.37, ${\text{η}}_{\text{p}}^{2}$ = 0.01] or severe stress symptoms [*F*(1, 122) = 0.06, *p* = 0.82, ${\text{η}}_{\text{p}}^{2}$ < 0.01]. This significant interaction is also reflected by the fact that participants with severe depressive symptoms only scored lower than participants without severe depressive symptoms before gameplay [*M_severe_* = 3.30, *SE_severe_* = 0.18; *M_non-severe_* = 3.76, *SE_non-severe_* = 0.07; *F*(1, 122) = 5.62, *p* < 0.05, ${\text{η}}_{\text{p}}^{2}$ = 0.04] and not after gameplay [*M_severe_* = 3.86, *SE_severe_* = 0.17; *M_non-severe_* = 3.87, *SE_non-severe_* = 0.06; *F*(1, 122) < 0.01, *p* = 0.96, ${\text{η}}_{\text{p}}^{2}$ < 0.01].

## Discussion

The current study examines an important, rarely addressed, factor in the potential success of therapeutic games: how we present and promote therapeutic games. With the number of interactive media interventions for mental health growing rapidly, it is vital to understand how these interventions are going to be accepted outside of research and clinical settings.

### Messaging and Game Choice

Our findings show that young adults with mild to severe mental health symptoms were almost four times more likely to select a game when it was explicitly promoted as beneficial for mental health compared to when it was promoted as entertaining. The most important conclusion we can draw from these results is that explicit mental health messaging did not deter young adults with mild to severe mental health symptoms from selecting a game, but in fact made the game more appealing to select. Overall, nearly 60% of participants selected the game they believed to be a mental health game. Given that regular professional help is sought by only approximately a quarter to a third of people with a diagnosable disorder (Alonso et al., 2004; Merikangas et al., 2011), it is encouraging that in a sample with elevated mental health symptoms three in five young adults showed interest in playing a game that purported to benefit their mental health.

Additionally, the results show that participants were almost six times more likely to select the game promoted with the mental health message in the detailed trailer as they were to select the mental health message in the abstract trailer. In our attempt to create two distinguishable trailers for the same video game we unintentionally created a version that was perceived as more attractive and fun. Although not manipulated as such, this incidental finding may suggest that both trailer messaging and trailer design are important factors in game choice. Accordingly, mental health games will likely be more successful if their trailer design is equally as appealing or even more appealing than the design of commercial videogames.

Furthermore, exploratory results show that symptom type did not predict game choice, suggesting that results are not specific to disorders and apply to internalizing issues in general. Interestingly, the same is found for symptom severity. Thus, youth with more severe symptoms were not more likely to turn to a mental health game, while most other studies indicate that people with severe symptoms are more likely to seek professional help (Merikangas et al., 2011; Sawyer et al., 2012). However, our result is in line with other studies that have shown that severity of depressive symptoms had no influence on help-seeking (Merikangas et al., 2011; Chin et al., 2015). Yet, considering that severe depressive symptoms have been found to decrease informal help-seeking (Sawyer et al., 2012; Chin et al., 2015), another explanation for our finding may lie in the undefined nature of mental health games. Mental health games may be interpreted as formal, informal or a separate category of help. This may have resulted in participants reacting to the therapeutic video game differently based on their interpretation. However, it is also possible that for mental health games, severity counteracts existing differences in help-seeking between those with more and less severe symptoms by decreasing barriers for help (Granic et al., 2014).

Moreover, exploratory analyses showed that the attractiveness and fun young adults expected of the games based on the trailers was not predicted by symptom severity or symptom type with the exception of anxiety symptoms. Participants with elevated anxiety symptoms rated both the entertainment and the mental health game as less fun based on the trailers than participants without anxiety symptoms. This may indicate that individuals more prone to anxiety are apprehensive of video games. Alternatively, a third variable (e.g., intolerance of uncertainty), which influences both the perceived fun of video games and the level of anxiety symptoms, may explain why participants with elevated anxiety symptoms expected the games to be less fun irrespective of messaging.

Thus, besides the influence of the trailer design the analyses provided no indication of why messaging differentially influenced participants. As two in five participants chose the entertainment game over the mental health game it is meaningful to explore why they may have made this decision and what may enhance the success of mental health messaging. First, participants may have experienced reactance or perceived stigma in response to the mental health trailer (e.g., one participant indicated that the mental health game suggested that there was ‘something wrong with me’) and therefore may have chosen the entertainment game instead. Previous research has shown that reactance may be limited both by warning people ahead of the persuasive intent of a health message (Richards and Banas, 2015) as well as by restoring their sense of autonomy after the message (Bessarabova et al., 2013). Yet, stigma is a broader societal problem, and evoking a sense of stigma cannot easily be avoided within an explicit mental health message.

Alternatively, participants may have selected the entertainment game to improve their mood in line with the mood management model (Zillmann, 1988) or to avoid potential mood damaging effects of the mental health game in line with the escape model (Perse, 1998; Oliver, 2009). Both motivations appear plausible. One participant explained her choice for the entertainment game indicating ‘If everybody likes it, it is very likely I will like it too’ supporting a mood management perspective. Whereas another participant argued the mental health game ‘looks frustrating and more complicated’ supporting an escape perspective. This suggests that explicit mental health games emphasizing positive game experiences would be even more attractive.

Finally, self-determination theory (Ryan and Deci, 2000) suggests participants may have chosen the entertainment game as the mental health game was not in line with their intrinsic motivations (e.g., one participant explained ‘I did not feel stressed or bad at the moment’). Though for some individuals beliefs about personal relevance may depend on their current state, suggesting that the mental health game may be selected at another point in time, others may never identify reducing stress as an intrinsic need. Thus, proclaiming to meet multiple needs may increase the chances of mental health games matching individual needs. Moreover, it will be valuable to further study motivations for mental health or entertainment game selection to inform strategies to attract even more young adults to mental health games.

### Game Experience

Besides testing the influence of messaging on game choice, the current study also looks at how the selected message influenced game experience. Young adults played Monument Valley for approximately 28 min regardless of game choice, mental health symptom severity or type. Additionally, as hypothesized, young adults who selected the mental health game experienced similar intrinsic motivation, autonomy and competence compared to those who selected the entertainment trailer. In contrast, exploratory analyses showed that although young adults who selected the entertainment message experienced similar autonomy, participants with severe symptoms who selected the mental health message felt more autonomous compared to participants without severe symptoms, confirming our expectation. In a similar vein but contrary to our expectations, participants with severe symptoms felt more competent than participants without severe symptoms, regardless of whether they believed the game was an entertainment or mental health game.

Finally, as expected, the current study shows that overall participants improved in affect over time, regardless of the message they selected. However, exploratory results suggest that this effect was driven by participants with severe depressive symptoms rather than those with no to moderate depressive symptoms or other severe symptoms. Thus, although we had expected participants with elevated depressive symptoms to experience more positive affect, the game increased positive affect only for those youth who reported severe depressive symptoms.

The current results replicate several of the findings from a previous study in which a mostly healthy sample of young adults were exposed to either a mental health or an entertainment-focused introduction message (Poppelaars et al., 2018). Both studies show that mental health messaging does not influence intrinsic motivation and affect. Both studies also show that positive affect increased for those with (severe) depressive symptoms, while there was no change in affect for those without (severe) depressive symptoms (i.e., using SAM). Perhaps most importantly, the current findings extend the previous one by indicating that these effects are particularly relevant for youth with severe depressive symptoms.

It is promising that those who are most affected by depressive symptoms show at least a short-term boost in positive affect. In general people with depressive symptoms report fewer positive experiences in their daily life (Peeters et al., 2003; Bylsma et al., 2011) and also have been shown to react with less positive affect to positive experiences in experimental settings (Bylsma et al., 2008). However, naturalistic studies show a mood-brightening effect in people with depressive symptoms, indicating that they may in fact be more sensitive to positive experiences in daily life and respond with more positive and less negative affect (Peeters et al., 2003; Bylsma et al., 2011). Thus, our results are in line with the mood-brightening effect in young adults with (severe) depressive symptoms. Temporary positive affect may partially explain why casual commercial games have been shown to reduce depressive symptoms (Russoniello et al., 2013). That is, according to the broaden-and-built theory, momentary positive emotions can create an upward spiral, broadening one’s perspective thus allowing one to seek out positive experiences, leading to more opportunities for positive emotions (Fredrickson, 2001). This upward spiral could eventually reduce depressive symptoms as depressed mood is replaced by a more positive mood.

Just like for mood, messaging had no effect on competence. However, exploratory analyses indicated that participants with more severe symptoms experienced enhanced competence following gameplay. This may reflect that youth with severe symptoms selected either the entertainment or the mental health game based on their capacity to deal with their issues and felt competent in the selection they made. Moreover, Nezlek and Gable (2001) propose that mental health issues are associated with unstable self-worth which increases sensitivity to daily events that may reflect on self-worth. As Monument Valley is designed for successful completion and triumphs are more visible (i.e., the avatar’s path becomes visible) than failures, participants with more severe symptoms may experience more pronounced competence after playing Monument Valley compared to participants with less symptoms and more stable self-worth.

The current study revealed no negative effect of mental health messaging on autonomy. These results may indicate that the ability to actively choose a game with a mental health message reduces the potential reactance evoked by the messaging. In fact, reactance to persuasive messages can be limited by providing a text confirming the choice freedom of individuals (Bessarabova et al., 2013), and the actual behavioral choice provided here may be even more successful in reestablishing a sense of choice freedom. Alternatively, we may hypothesize that the participants who felt that the mental health message was controlling and who would have therefore experienced less autonomy in the game, selected the entertainment game and so avoided experiencing less autonomy. Additionally, both explanations could work in unison.

Moreover, exploratory analyses indicated that when the mental health game was selected, participants with severe mental health symptoms experienced more autonomy than participants without severe symptoms. Thus, suggesting that mental health messaging can stimulate a sense of autonomy. Possibly, participants with severe mental health symptoms felt the mental health aim to be especially in line with their own values and motivations, thus leading to an enhanced sense of autonomy when choosing this option. However, this did not result in the expected accompanying increase in intrinsic motivation.

### Strengths, Limitations, and Future Directions

Besides focusing on a societally relevant research question, the current study has some additional strengths. First, our design is an important strength to highlight. We provided participants with a controlled choice between an explicit mental health and a stealth entertainment message that in reality promoted the same game. Participants were able to freely choose and play the game for as long as they liked (within the study’s time-constraints) creating a genuine game experience and an objective measure of engagement, avoiding self-report biases that come with post-game questionnaires. Second, the counterbalanced presentation of the two trailer designs in which the messages were shown allowed us to separate the effects of messaging from the perceived attractiveness of the trailers. Third, we could relate any differences in game play experiences to messaging because all players played the same commercial game and because there was no contamination of any in-game mental health content, as would be the case with most existing therapeutic games. Finally, the current study included at-risk participants with elevated mental health symptoms, arguably the most relevant target audience for therapeutic games. Thus, our sample is likely representative of the audience who would be seeking such games and serious game designers with mental health targets would benefit from incorporating our messaging results.

Naturally, the study also has a number of limitations. The sample overrepresented highly educated young adults, females, and social science students due to our recruitment strategy. Highly educated social science students may be more open to and curious about innovative mental health interventions, which may have inflated the interest for the mental health game. Similarly, women may potentially be more attracted to mental health games than men, as a recent review indicates that gender influences gaming motives and behavior (Veltri et al., 2014), which could have skewed our results. Thus, it is advisable for future research to include men and women equally, to target a more diverse group of young adults, as well as to target other age groups as children may react differently to messaging.

Another limitation to consider is the experimental setting in which participants selected and played the game. First, the length of the experiment limited gameplay to a single session under 45 min. Vital differences in gameplay patterns between mental health and entertainment games may only become visible when repeated gameplay is possible and sessions are not artificially limited. Therefore, future research is needed that observes naturalistic gameplay over a matter of weeks or even months.

Second, participants may still have selected a different game than they would have outside the lab due to several experimental factors. The choice within the experiment was limited to two games, rather than the reality of almost unlimited video game choice, in which one mental health game may not even attract enough attention to be considered as an option. Moreover, participants may have behaved in a socially desirable way when choosing a game, knowing that their choice was recorded. On the one hand, participants may have assumed that selecting the mental health game (i.e., an uncommon game type) was preferred by the experiment leader. On the other hand, participants may have felt that selecting the mental health game indicated needing a therapeutic intervention and therefore selected the more normative entertainment game. A final way the experimental setting may limit the generalizability of this study is the fact that the experimental environment may have encouraged participants to make a more thoughtful choice, potentially anticipating that they would have to explain their choice. In everyday situations, game choice may be a more instinctive choice (i.e., based on unconscious decision processes) as well as a more implicit choice (i.e., multiple games may be played and selecting game X does not rule out playing game Y).

Consequently, future research exploring more promotion channels for therapeutic games and the relative success of explicit and stealth messaging will be valuable. Youth can be recommended video games through a myriad of sources such as friends, blogs, forums, online video game stores (e.g., Steam), video game news and review sites and popular video game players who demonstrate games online (e.g., on YouTube or Twitch). Research on any of these sources of recommendations can help clarify if messaging may need to be adapted per situation. Additionally, alternative messaging may be explored. For example, messaging may combine entertainment and mental health messaging in various proportions or alternatively explicit messaging may describe causes of mental health issues rather than the effects of the game. A recent study shows that young adults believed a mental health app to be more useful and had higher intentions to use it if a prior message emphasized internal causes of depression (Khan and Peña, 2017).

Furthermore, in real-life youth may only play a video game after receiving recommendations from multiple sources (e.g., a friend, review site and popular gamer recommending the same game) and future research may study this complexity (see Konijn et al., 2013 for methods to use YouTube for research purposes). In order to enhance the generalizability of the research results further, researchers may provide more choice to youth. Certainly, if youth are presented with an entire webpage of video games, which may easily contain 50 video games, the chances of them selecting a single mental health game will be lower than the almost 60% found in the present study. However, given the fact that youth would not necessarily only play one game recommended on such a page, it will be critical to see if youth will consider mental health games in such a scenario and what aspects influence the likelihood for youth to play therapeutic games.

In addition to messaging, individual characteristics besides symptoms may also influence game choice and experience. The current study did not assess if participants were diagnosed with a mental health disorder nor if they themselves believed that they had a mental health disorder. Participants who are aware of a mental disorder may be more likely to select a mental health game as they feel that is meant for them in comparison to participants who do not identify their symptoms as a coherent mental issue. Additionally, game choice is likely affected by personal motivational factors, the individual’s believes concerning the value of video games and their beliefs about mental health disorders. For example, someone who considers video games violent and a waste of time may be much less likely to find a mental health game credible than someone who believes video games can be educational and foster social connections. Similarly, the stigma that one experiences surrounding mental health or the extent to which one believes mental disorders cannot effectively be treated can limit the effectiveness of mental health messages. Thus, future research may examine which individual factors influence the choice between explicit and stealth messaging.

Finally, future research will need to study the effects of messaging on game effectiveness. Naturally, games that are not played cannot be effective. However, while maintaining the attractiveness of the game, the promotion may also aim to enhance effectiveness. Intrinsic motivation for playing a game may enhance its effectiveness. Furthermore, explicit mental health messaging promising health benefits may enhance the effectiveness of therapeutic games through the positive expectations of the player (Enck et al., 2013).

## Conclusion

Therapeutic games, other serious games (e.g., educational games) and other self-administered interventions will have the biggest impact if they are widely available and can motivate people to seek out and stay engaged in these interventions. Thus, it is critical to consider how messaging in the promotion of therapeutic games can attract youth and support an immersive game experience. The current study indicates that once effective therapeutic games targeting youth outside of a clinical setting are developed, these games may indeed be attractive to youth, especially when promoted in an engaging trailer that includes an explicit mental health message.

This study has several implications for the design of games that target a wide range of mental health issues and the messaging used in these games. First, as youth with the most severe symptoms in our study were found to have a better game experience in terms of autonomy, competence, and affect, special attention needs to be paid to youth with mild to moderate symptoms. When designing therapeutic games for youth with mild to moderate symptoms, supporting autonomy and competence either in game design or in promotional messaging is especially important, given that these factors predict a more positive game experience (Ryan et al., 2006). Second, designers of therapeutic games may search for ways to improve mood for youth without severe depressive symptoms to enhance engagement. This is especially important if the game’s effectiveness relies partially on short-term increases in mood. Finally, this study indicates that elevated anxiety symptoms were related to expecting less fun from video games. Thus, it may valuable for those developing anxiety reducing therapeutic games to study if promoting these games in a different way makes them appear more fun and so allows youth to take the initiative to play therapeutic games.

Many research questions remain regarding the promotion of therapeutic games, but so far the results support the idea that explicitly marketing games as beneficial to mental health may not turn youth away from these games. Instead, they may provide youth with the opportunity to improve their well-being in an autonomous and engaging manner.

## Ethics Statement

This study was carried out in accordance with the recommendations of the Code of Ethics for Research in the Social and Behavioural Sciences Involving Human Participants, the ethical committee of the Faculty of Social Sciences at Radboud University. The protocol was approved by the ethical committee of the Faculty of Social Sciences at Radboud University (ECSW2017-3001-461). All subjects gave written informed consent in accordance with the Declaration of Helsinki.

## Author Contributions

MP, AW, AL-A, and IG contributed to the conception and design of the study and discussed the interpretation of the results. MP developed the trailer design and messaging with feedback from AW, AL-A, and IG. MP and AW organized and performed the data collection and responsible for data management. AW coordinated the data collection. AL-A and IG supervised the project. MP performed the statistical analyses and wrote the first draft of the manuscript. All authors contributed to manuscript revision, read and approved the submitted version.

## Conflict of Interest Statement

The authors declare that the research was conducted in the absence of any commercial or financial relationships that could be construed as a potential conflict of interest.
